# Supplementary material for: “It reminds me and motivates me”: Human-centered design and implementation of an interactive, SMS-based digital intervention to improve early retention on antiretroviral therapy: Usability and acceptability among new initiates in a high-volume, public clinic in Malawi
Source: PLoS One. 2023 Jul 20;18(7):e0278806. doi: 10.1371/journal.pone.0278806 (PMC10358959; doi:10.1371/journal.pone.0278806)
Supplement: S3 File — Qualitative short-answer responses from usability survey (English). (PDF) [file pone.0278806.s003.pdf]

General comment

1. A good system. It reminds me of clinic appointments
2. Add more messages to cover some of the challenges we face
3. All is well. I asked question on 3044 but I was not answered properly and was answered when I asked another question. Try to answer us quickly
4. Although the messages do not have information about ARVs, I am afraid that some who receives similar messages may disclose my status. The messages are good as I am reminded of my clinic appointment date whenever I am busy
5. Am always afraid that someone might see the messages as my phone is always with neighbors. Someone who knows the number 3044 might realise that this message is from Lighthouse and might compromise with my privacy.
6. Am always in the field travelling and doing business and these places am in most of the times there are no electricity and with that in mind I always leave the phone at home and that's the reason why I don't reply to the messages.
7. Am always afraid of someone sees my messages is going to ask me where the messages are coming from. That also contributed for me to stop the messages, especially the message about having three days or one day to come to the clinic.
8. Continue offering the service as it reminds me of my visit date to the clinic
9. Continue sending messages as they are encouraging us to stay health. It reminds me and motivates me.
10. Continue sending us messages. Most people forget their clinic appointment
11. Continue sending us motivational messages and visit reminder messages as these messages shows that were are not discriminated and shows that there are many people with problems
12. Continue this arrangement as it reminds me of my appointment dates
13. Continue what you have started. I am able to inform the clinic that I travelled. I appreciate this program sometimes I am busy so the messages remind me of the appointment
14. Do not stop sending messages as I am encouraged. But do not forget to greet me
15. Do not stop sending messages as this is a good arrangement
16. I am encouraged through these messages so do not stop
17. I am thankful that you remind and encourage us
18. I am thankful that you send reminder visit messages. Through these interactions, we are no afraid as we know you will help us
19. I am very thankful when I receive these messages
20. I can recommend the system to other people because it helps me a lot in remembering my visit days.
21. I have been failing to talk to healthcare workers about my health so this approach has helped me connect with healthcare workers
22. I receive these messages through my wife phone number. I do not respond to some texts messages because I stay far but she informs me when I come back
23. I recommend the team for not including in the messages any word about ARVs.
24. I usually forget my appointment date so the system reminds me. I have a friend who wants to receive similar messages
25. I would like to appreciate the system, it reminds me of my visit date and sometimes to take medicines. It also helps to prepare health passport and transport and it also reminds me about my status.
26. I would like to encourage you to continue sending these messages. Phone call risks disclosing our status especially when you receive it amidst friends

27. I would like to request you to continue as some of us easily forget
28. I would recommend the visit reminders to friends because I think they can help a lot people who forget dates.
29. If you forget your days you are reminded and we feel being helped and continue offering this service.
30. It is good and I am encourage; I reminded to take medication and visit the clinic
31. It's a good arrangement as I am reminded of ARVs and encouraged
32. It's a good arrangement for those who frequently forgets
33. It's a good idea as you Lighthouse team you keep on checking on us. Continue this good cause.
34. It's a good system and it is working for me and continue.
35. It's good and does not disturb me because its does not require a lot of text
36. It's good, continue. It encourages us
37. Messages are very good and encourage me. Continue sending the messages
38. Messages help me because I remember the appointment dates
39. My phone was sold by my wife but please continue sending these messages, I will buy another phone soon.
40. No comment
41. None
42. She is happy with the study. forgot about her visit today but because of the visit reminder message she managed to come.
43. She loves the study especially the provisions to interact with the study team whenever she wants to.
44. SMS visit reminder is better than through phone call because colleagues asks us why someone is calling me
45. Some messages need to come out clear but otherwise continue sending them. You have to include more messages that remind us about what was discussed before ART initiation as a reminder
46. Thank you as your message encourages us about good health and to take medication. The program is good and scale it up to many people as some people require these reminders
47. The messages are good because he is at work but the moment he receives it reminds him of coming him at the clinic.
48. The messages are fine and a big reminders to my visits
49. The messages are good as I am able to prepare transport in advance. Continue sending them
50. The messages are good as they remind me of the clinic appointment and good health
51. The messages are good because they encourages us to go to the hospital on our appointment date
52. The messages are good because they give us the reminder for the visits
53. The messages are good because they remind me of the clinic appointment
54. The messages are good for people who know how to read. You should also prepare something for those who can not read
55. The messages are good. We are reminded that we have three days to go to the clinic
56. The messages are helpful as I am reminded of my clinic appointment and about my health
57. The messages are helpful, continue sending them. If I travel far. I am able to plan for my clinic visit by returning home

58. The messages are simple and easy to understand and they do remind me of my visits which is important. I LOST my phone and it has been a month since I received the messages but they have been so important.
59. The messages help me when I forget my clinic appointment
60. The system is good it remind me about the visit and to prepare.
61. The text messages are helpful
62. 'The text messages are helpful as they remind us of the clinic appointments
63. 'The text messages are helpful as they remind us of the clinic appointments. Also, we are able to change the appointment date
64. These messages are good because they remind me the visits because most of the times I do forget.
65. This is a very good service and the messages are very special.
66. This system is good and continue offering us this service. We got reminded when we forget the dates. I came on 26 January for consultation but I didn't get drugs then when my days were coming close I received the reminder message. I thought in my mind that I had already received the meds. Thank you for reminding me
67. This system is very important as it reminds me of my visit date and motivates me to take drugs. This system also is helpful because it also reminds us when we have missed our visit.
68. Try to do more to others, this messages are helping a lot. Large number of people do forgot their dates this system will help in reminding about their scheduled date and might help in bringing back those who could have never come back.
69. Very thankful for the messages that she receives especially visit reminders and the way are treated when we come to the clinic. We are very thankful.
70. We are being helped a lot with the messages because at times I do forget to check from the health passport book.
71. When I see the messages I do have the conscious to take the medicine.
72. (blank)
73. Messages remind me to take ARVs. I have even told my friends and my wife to inform me when they see a message from 3044 number. I would be happy if I receive visit reminder messages from 7 days before my appointment
74. The messages remind me of the clinic appointment and to take nutritious food
75. The messages remind me of the clinic appointment and shows that LH would like to help us
76. The messages remind me of the clinic appointment when I forget
77. The messages are encourage and remind me of the clinic visits so continue sending them and scale up the services to other people
78. Continue as the messages remind me of the clinic visit
79. Messages remind me as I sometimes forget
80. I am thankful because the messages remind me of my clinic appointment and encourages me to live a health life
81. The messages remind me whenever I am busy with other things
82. I am sometimes busy so I forget the clinic appointment. The messages remind me about the clinic appointment
83. Sometimes I forget the clinic appointment so the messages remind me
84. The messages remind me of the appointment date
85. The messages remind us of the appointment date especially for those who quickly forget

86. The messages encourage me and my wife and remind me of the clinic appointment when I forget
87. The messages encourage me and remind me of the clinic appointment. Thank you. Continue sending the messages
88. I am grateful because all is well as the messages remind us

Improvements on motivation messages

1. Add messages that motivate a person to get tested. The messages should be open and should include ART words
2. Add messages about nutrition
3. I am afraid that when people see the message Stop would ask me what I want to stop
4. I am confused with the message stop
5. I am confused with the message stop and do not know what to do
6. I get confused when I see the message stop so I do not reply to the messages because I do not want to stop
7. Messages should come as an advise not instruction
8. Mostly the messages you are sending are vague and broad and they are not specific. For example the messages that tells us about our health being good while maybe we are sick.
9. Nothing
10. Send different messages in a week
11. Send messages before we wake up
12. Send more messages
13. Send these messages on Friday as we are not busy on this day.
14. Some messages are not meant for me as a widow. If you can make the messages as general as possible.
15. Sometimes we want you to be replying our messages about our health. If we ask you about our personal health or which drugs to take or buy we would love if you reply to us in a timely manner
16. These text messages are good and ensures privacy
17. You confuse us because you always tell us to write stop
18. You greet us

Improvements on visit reminder messages

1. Can you work on messages that I sent after missing a clinic appointment
2. Changing visit date after I have already indicated that I am coming
3. I want to receive them in English
4. Nothing
5. Send the messages before bedtime
6. Send the messages before we wake up
7. Stop sending messages reminding us that we have missed the visit when we already reported.
8. You can change to another language if I chose Chichewa
