# Supplementary material for: “It reminds me and motivates me”: Human-centered design and implementation of an interactive, SMS-based digital intervention to improve early retention on antiretroviral therapy: Usability and acceptability among new initiates in a high-volume, public clinic in Malawi
Source: PLoS One. 2023 Jul 20;18(7):e0278806. doi: 10.1371/journal.pone.0278806 (PMC10358959; doi:10.1371/journal.pone.0278806)
Supplement: S4 File — (DOCX) [file pone.0278806.s004.docx]

**Client Satisfaction Survey for SMS follow-up for retention**

Section 1: Background

1.Study ID#________________________

2. Patient has been a 2wT client for how long? Circle one: 3-months 6-months

3. Date:________________________

4. Has client received any motivational messages (circle one)? Yes No

5. Has client received any visit reminders? (circle one)? Yes No

*******IF RESPONSES TO BOTH Q4 AND Q5 ARE “NO,” STOP HERE. THANK CLIENT.**

**Section 2: 2wT usability assessment**

*Hi [client name]. We are trying to make the system better for the patients, like you. To do this, we are trying to make sure that the directions and education are as clear as they can be. I want to ask you about how to use the system so that I can understand where we can make the system better. I would like to ask you now about how you would respond to some messages. I would also like you to please tell me if something is not clear. I will listen closely so that we can make the system better.*

*I am going to show you 4 pictures. Then, I will ask you what you would do. There is no right or wrong answer.* Interviewer takes notes on how system is confusing or how it can be improved:

For interviewer, did client complete the following tasks correctly:

6. How to confirm visit attendance: picture 1 (circle one)? Yes No

7. How to transfer: picture 2 (circle one)? Yes No

8. How to change visit date: picture 3 (circle one)? Yes No

9. Again: how to change visit date: picture 4 (circle one)? Yes No

***Please note any issues in client’s confusion or suggestions for how to make the system better:

___________________________________________________________________________________________________________________________________________________________________________________________________________________________________________________________________________________________________________________________________________________________________________________________________________________________________________________

**Section 3. 2wT usability and acceptability assessment**

*Now, I would like to ask your opinion about the 2wT study and those SMS you received from us. For each of these* ***questions****, we’d like to respond, “yes” “no” or “Don’t know/no response”.*

**Directions:** Please **CIRCLE ONE** answer for each question.

*Hi [client name]. I would like to ask your opinion about the 2wT study and those SMS you received from us. For each of these* ***questions****, we’d like to respond, “yes” “no” or “Don’t know/no response”.*

|  |  | RESPONSE | | |
| --- | --- | --- | --- | --- |
| 10 | Did you understand what SMS would arrive on your phone each week (motivational messages)? | Yes | No | DK/No response |
| 11 | Were you clear on how to respond to the visit reminders? | Yes | No | DK/No response |
| 12 | Did you like receiving supportive messages each week? | Yes | No | DK/No response |
| 13 | Do you think weekly messages helped you take your meds? | Yes | No | DK/No response |
| 14 | Did you like the visit reminders? | Yes | No | DK/No response |
| 15 | Did you have challenges responding to visit reminders? | Yes | No | DK/No response |
| 16 | Did you have challenges receiving texts? | Yes | No | DK/No response |
| 17 | Were you afraid someone else would see motivational texts? | Yes | No | DK/No response |
| 18 | Were you afraid someone else would see visit reminders? | Yes | No | DK/No response |
| 19 | Did you feel that someone at Lighthouse was supporting you? | Yes | No | DK/No response |
| 20 | Were you worried about privacy receiving these texts? | Yes | No | DK/No response |
| 21 | Did the messages help you remember your visit date? | Yes | No | DK/No response |
| 22 | Was it easy to change your visit date using text? | Yes | No | DK/No response |
| 23 | Were you satisfied with the motivational messages? | Yes | No | DK/No response |
| 24 | Were you satisfied with the visit reminder messages? | Yes | No | DK/No response |
| 25 | Would you recommend motivational messages to friends? | Yes | No | DK/No response |
| 26 | Would you recommend reminder messages to friends? | Yes | No | DK/No response |

| 27 | How often would you like to receive motivational or supportive messages? | Daily | Weekly | Never | Other |
| --- | --- | --- | --- | --- | --- |
| 28 | How could we improve the motivational supportive texts? |  | | | |
| 29 | How could we improve the visit reminders? |  | | | |
| 30 | How could the texting follow-up be improved? |  | | | |
| 31 | Any other comments for the study team? |  | | | |
